# Self-rated health (SRH) partially mediates and associations between personality traits and life satisfaction in older adults

**DOI:** 10.3389/fpsyg.2023.1189194

**Published:** 2023-07-06

**Authors:** Weixi Kang, Antonio Malvaso

**Affiliations:** ^1^Imperial College London, London, United Kingdom; ^2^University of Pavia, Pavia, Lombardy, Italy

**Keywords:** personality, self-rated health, life satisfaction, mediation, older adults

## Abstract

It is established that personality traits contribute to life satisfaction but why they are connected are far less understood. This research report tested if self-rated health (SRH) which is one’s subjective ratings of their health and has a high predictivity of actual health mediates the associations between the Big Five model of personality and life satisfaction in a cohort (*N* = 5,845) of older adults from the UK. By using Pearson’s correlation analysis and mediation analysis, the current research reported positive correlations between Agreeableness, Openness, Conscientiousness, and Extraversion, SRH, and life satisfaction. However, Neuroticism was negatively correlated with SRH and life satisfaction. The main findings were that SRH partially mediates the associations between all traits in the Big Five and life satisfaction in older adults. This study began novel exploration on if SRH could explain the connections between the Big Five and life satisfaction. Results revealed SRH could partially explain these associations in all traits. These results may offer additional support to recently developed integrated account of life satisfaction, which argues that there are no single determinants of life satisfaction. Rather, life satisfaction is made up by many factors including but not limited to personality and health.

## 1. Introduction

As the cognitive aspect of subjective well-being (SWB), life satisfaction refers to an individual’s overall subjective evaluation or perception of their own life as a whole. Understanding the contributing factors of life satisfaction can lead to a better comprehension of the broad sense of SWB (Schimmack et al., 2002). Indeed, in the past several decades, several theoretical models have been developed to account for life satisfaction mainly including the bottom-up (Headey et al., 2005; Diener, 2009; Erdogan et al., 2012; Loewe et al., 2014) and top-down (Headey et al., 2005; Diener, 2009; Erdogan et al., 2012; Loewe et al., 2014) model. Specifically, the bottom-up model suggests that life satisfaction is a sum of satisfaction of aspects of life including but not limited to satisfaction with health, income, and housing. The top-down perspective of life satisfaction proposes personality traits can decide life satisfaction. These two theories do not seem to be controversial but can be integrated. Indeed, during the past few years, empirical evidence seems to favor an integrated account of life satisfaction, which argues that life satisfaction is made up of satisfaction towards aspects of life and personality traits (Lachmann et al., 2017; Malvaso and Kang, 2022).

Thus, according to these theories, personality traits and health are all relevant to life satisfaction. The Big Five is a widely used inventory that measures personality traits including Openness, Extraversion, Agreeableness, Conscientiousness, and Neuroticism. The Big Five has been consistently found to be associated with health (Strickhouser et al., 2017) such as biological markers (Stephan et al., 2018a,b), mental health (Hakulinen et al., 2015a; Kang et al., 2023), risks of chronic conditions such as Alzheimer’s disease (Terracciano et al., 2014) and mortality risk (Graham et al., 2017). Self-rated health (SRH) refers to the subjective rating of one’s own health, which is strongly predictive of actual healthy including risks of chronic disease, cognitive decline, dementia, and mortality (DeSalvo et al., 2006; Montlahuc et al., 2011; Latham and Peek, 2013; Bendayan et al., 2017).

Regarding the specific directions of the relationships between the Big Five, SRH, and life satisfaction, Neuroticism tends to be negatively related to SRH (Chapman et al., 2006; Löckenhoff et al., 2012; Turiano et al., 2012; Kööts-Ausmees et al., 2016; Mund and Neyer, 2016; Stephan et al., 2020). Openness (Löckenhoff et al., 2012; Stephan et al., 2020), Extraversion (Goodwin and Engstrom, 2002; Löckenhoff et al., 2012; Turiano et al., 2012; Stephan et al., 2020), and Conscientiousness (Löckenhoff et al., 2012; Turiano et al., 2012; Stephan et al., 2020; Kitayama and Park, 2021) tend to have a positive link to SRH although some studies did not find such a connection between Openness and SRH (Turiano et al., 2012; Kööts-Ausmees et al., 2016; Mund and Neyer, 2016; Stephan et al., 2020). The connection between Agreeableness and SRH remains still controversial (Löckenhoff et al., 2012; Turiano et al., 2012; Stephan et al., 2020). Similarly, literature generally found that Neuroticism has a negative relationship with life satisfaction whereas Agreeableness, Openness, Extraversion, and Conscientiousness have a positive connection to life satisfaction (e.g., Lachmann et al., 2017; Malvaso and Kang, 2022). Finally, SRH is strongly associated with life satisfaction regardless of country and age (e.g., Kööts-Ausmees and Realo, 2015; Atienza-González et al., 2020).

Given these pieces of evidence, there is good reason to believe that the Big Five personality traits may contribute to life satisfaction via SRH pathways. Indeed, personality traits can contribute to objective health such as sleep (Stephan et al., 2018a,b), physical activities (Sutin et al., 2016), and inflammation (Luchetti et al., 2014) and addictive behaviors (Kang, 2022a,b, 2023) that may ultimately affect health and are associated with SRH. Moreover, as speculated by prior studies (e.g., Lachmann et al., 2017; Malvaso and Kang, 2022), being healthy is one of the most important factors that people use to assess life satisfaction. Importantly, this may become a more critical factor in evaluating life satisfaction in older adults, who are experiencing a lot a more health issues that may interfere with their daily life than their younger counterparts.

Thus, the current research report aimed to investigate if the Big Five are related to life satisfaction via SRH pathway in older adults. The current research hypothesizes that SRH meditates the relationships between the Big Five and life satisfaction.

## 2. Methods

### 2.1. Data

Wave 3 data from Understanding Society: the UK Household Longitudinal Study (UKHLS) were collected between 2010 and 2011 and were selected for this study (University of Essex, 2022). Please refer to https://www.understandingsociety.ac.uk for more information. Wave 3 was used because only Wave 3 contains assessments of all main variables of interest. Data collections have been approved by the University of Essex Ethics Committee. People who were younger than 65 years old or have missing variables were removed, which resulted in a sample of 5,845 older adults.

### 2.2. Measures

#### 2.2.1. Personality traits

UKHLS Wave 3 used the 15-item version of the Big Five, which has been shown to be a valid version to measure the Big Five with good internal consistency, test–retest reliability, convergent validity, and discriminant validity (Hahn et al., 2012; Soto and John, 2017). Please refer to Supplementary material for a complete set of these questions. A Likert scale ranging from 1 (“disagree strongly”) to 5 (“agree strongly”) were used to score these responses. This study reverse-coded some of scores when appropriate.

#### 2.2.2. SRH

SRH was captured by the question “In general, would you say your health is…” using a 5-point Likert scale from 1 (excellent) to 5 (very poor). Responses from this question were reversed coded so a higher score reflects better health. The reliability of it is high (e.g., Lundberg and Manderbacka, 1996).

#### 2.2.3. Life satisfaction

Life satisfaction was measured by the question “How dissatisfied or satisfied are you with… your life overall?” Participants answered this question with a 7-point scale ranging from 1 (not satisfied at all) to 7 (completely satisfied). The results of single-item measure are pretty similar to multi-item inventories such as the Satisfaction with Life Scale (SWLS; Cheung and Lucas, 2014).

#### 2.2.4. Study control variables

Age, sex, income (monthly), education, and marital status were included as study control variables. The coding of these can be found in Table 1.

**Table 1:** Descriptive statistics of demographic characteristics, personality traits, and SRH.

Variable	Mean	S.D.	N	%
Age	72.93	6.38	0.79	3.00
Monthly income	1314.66	1296.88	0.75	9.38
Neuroticism	3.16	1.46	0.43	5.69
Agreeableness	5.74	1.04	−0.77	3.55
Openness	4.36	1.42	−0.24	2.67
Conscientiousness	5.51	1.18	−0.56	2.89
Extraversion	4.54	1.38	−0.12	2.62
SRH	3.08	1.12	−0.12	2.23
Life satisfaction			−1.48	4.76
Sex				
Male	2,726	46.64		
Female	3,119	53.36		
Highest educational qualification				
Below college	4,873	83.37		
College	972	16.63		
Legal marital status				
Single	2,325	39.78		
Married	3,520	60.22		

## 3. Analysis

Pearson’s correlation coefficients (r) were first calculated between personality traits including Agreeableness, Openness, Extraversion, Neuroticism, and Conscientiousness, SRH, and life satisfaction. Mediation analysis is a statistical method used to examine the mediating effect of a variable in explaining the relationship between two other variables. It aims to determine whether and how much of the relationship between an independent variable (X) and a dependent variable (Y) can be explained by the influence of a mediator variable (Baron and Kenny, 1986). Mediation analyses were conducted using the mediation toolbox on MATLAB 2018a by setting personality traits as the independent variables, SRH as the mediator, and life satisfaction as the dependent variable with 10,000 bootstrap sample significance testing with study control variables as covariates.^1^

## 4. Results

Descriptive statistics were demonstrated in Table 1. Pearson’s correlation revealed that Neuroticism is negatively correlated with SRH [*r* = −0.18, *p* < 0.001, 95% C.I. (−0.21, −0.16)] and life satisfaction [*r* = −0.22, *p* < 0.001, 95% C.I. (−0.25, −0.19)]. Agreeableness had correlations with SRH [*r* = 0.04, *p* < 0.001, 95% C.I. (0.01, 0.06)] and life satisfaction [*r* = 0.11, *p* < 0.001, 95% C.I. (0.09, 0.14)]. The correlations between Openness and SRH [*r* = 0.13, *p* < 0.001, 95% C.I. (0.11, 0.16)] and life satisfaction [*r* = 0.08, *p* < 0.001, 95% C.I. (0.06, 0.11)] were positive. Conscientiousness was positively correlated with SRH [*r* = 0.17, *p* < 0.001, 95% C.I. (0.14, 0.19)] and life satisfaction [*r* = 0.17, *p* < 0.001, 95% C.I. (0.14, 0.19)]. Extraversion had positive associations with SRH [*r* = 0.05, *p* < 0.001, 95% C.I. (0.03, 0.08)] and life satisfaction [*r* = 0.10, *p* < 0.001, 95% C.I. (0.08, 0.12)]. Finally, SRH was positively correlated with life satisfaction [*r* = 0.25, *p* < 0.001, 95% C.I. (0.23, 0.27)] (see Table 2).

**Table 2:** Pearson’s correlation coefficients (r) between Neuroticism, Agreeableness, Openness, Conscientiousness, SRH, and life satisfaction.

Variables	Neuroticism	Agreeableness	Openness	Conscientiousness	Extraversion	SRH
Agreeableness	−0.09 ^***^					
Openness	−0.10 ^***^	0.17 ^***^				
Conscientiousness	−0.15 ^***^	0.32 ^***^	0.25 ^***^			
Extraversion	−0.13 ^***^	0.18 ^***^	0.23 ^***^	0.20 ^***^		
SRH	−0.18 ^***^	0.04 ^***^	0.13 ^***^	0.17 ^***^	0.05 ^***^	
Life satisfaction	−0.22 ^***^	0.11 ^***^	0.08 ^***^	0.17 ^***^	0.10 ^***^	0.25 ^***^

^***^p < 0.001.

**Figure 1:**
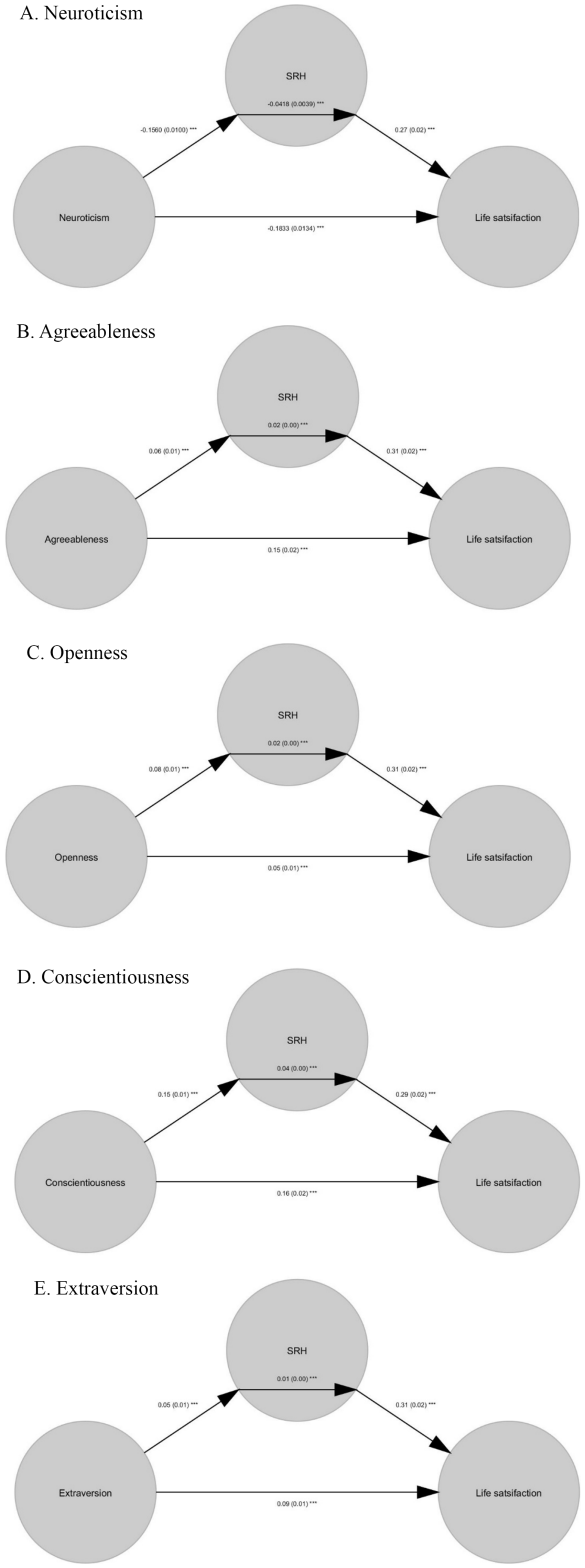
The path diagram for **(A)** Neuroticism, **(B)** Agreeableness, **(C)** Openness, **(D)** Conscientiousness, and **(E)** Extraversion. Standardized coefficients are included in the parenthesis.

Mediation analysis found that SRH partially mediated the relationship between Neuroticism [mediation effect: *b* = −0.04, *p* < 0.001, 95% C.I. (−0.04, −0.04); direct effect: *b* = −0.18, *p* < 0.001, 95% C.I. (−0.19, −0.17)], Agreeableness [mediation effect: *b* = 0.02, *p* < 0.001, 95% C.I. (0.02, 0.02); direct effect: *b* = 0.15, *p* < 0.001, 95% C.I. (0.14, 0.16)], Openness [mediation effect: *b* = 0.02, *p* < 0.001, 95% C.I. (0.02, 0.03); direct effect: *b* = 0.05, *p* < 0.001, 95% C.I. (0.04, 0.06)], Conscientiousness [mediation effect: *b* = 0.04, *p* < 0.001, 95% C.I. [0.04, 0.05]; direct effect: *b* = 0.16, *p* < 0.001, 95% C.I. (0.15, 0.17)] and Extraversion [mediation effect: *b* = 0.01, *p* < 0.001, 95% C.I. (0.01, 0.02); direct effect: *b* = 0.09, *p* < 0.001, 95% C.I. (0.08, 0.10)]. The complete results were demonstrated in Table 3 and Figure 1.

**Table 3:** The median analysis results for A. Neuroticism, B. Agreeableness, C. Openness, D. Conscientiousness, and E. Extraversion.

A. Neuroticism					
	a	b	c’	c	ab
Coeff	−0.16	0.27	−0.18	−0.23	−0.04
STE	0.01	0.02	0.01	0.01	0.00
t (~N)	−15.54	15.66	−13.67	−16.57	−10.83
Z	−3.73	3.72	−3.69	−3.71	−3.68
CI lb	−0.16	0.26	−0.19	−0.23	−0.04
CI ub	−0.15	0.28	−0.17	−0.22	−0.04
p	0.0002	0.0002	0.0002	0.0002	0.0002

B. Agreeableness					
	a	b	c’	c	ab
Coeff	0.06	0.31	0.15	0.17	0.02
STE	0.01	0.02	0.02	0.02	0.00
t (~N)	4.01	18.16	8.07	8.74	3.90
Z	3.74	3.73	3.72	3.74	3.77
CI lb	0.05	0.30	0.14	0.16	0.02
CI ub	0.07	0.32	0.16	0.18	0.02
p	0.0002	0.0002	0.0002	0.0002	0.0002

C. Openness					
	a	b	c’	c	ab
Coeff	0.08	0.31	0.05	0.08	0.02
STE	0.01	0.02	0.01	0.01	0.00
t (~N)	7.74	17.86	3.69	5.33	7.05
Z	3.71	3.73	3.72	3.71	3.77
CI lb	0.07	0.30	0.04	0.07	0.02
CI ub	0.09	0.32	0.06	0.09	0.03
p	0.0002	0.0002	0.0002	0.0002	0.0002

D. Conscientiousness					
	a	b	c’	c	ab
Coeff	0.15	0.29	0.16	0.20	0.04
STE	0.01	0.02	0.02	0.02	0.00
t (~N)	12.37	16.89	9.65	12.09	9.87
Z	3.74	3.72	3.70	3.69	3.76
CI lb	0.14	0.28	0.15	0.19	0.04
CI ub	0.16	0.30	0.17	0.21	0.05
p	0.0002	0.0002	0.0002	0.0002	0.0002

E. Extraversion					
	a	b	c’	c	ab
Coeff	0.05	0.31	0.09	0.11	0.01
STE	0.01	0.02	0.01	0.01	0.00
t (~N)	4.30	18.21	7.30	8.20	4.20
Z	3.70	3.73	3.69	3.67	3.72
CI lb	0.04	0.30	0.08	0.10	0.01
CI ub	0.05	0.32	0.10	0.12	0.02
p	0.0002	0.0002	0.0002	0.0002	0.0002

## 5. Discussion

This research report sought to test whether SRH mediates the associations between the Big Five and life satisfaction in older adults. Results provided novel findings that SRH partially mediates all the associations between the Big Five and life satisfaction.

The finding that SRH mediates the relationship between Neuroticism and life satisfaction is totally explainable given that Neuroticism is tendency to experience negative emotions, which is associated with affective disorder (Hakulinen et al., 2015a) and other chronic diseases including dementia (Terracciano et al., 2014) and chronic respiratory diseases (Terracciano et al., 2017). In addition, Neuroticism is positively connected with addictive behaviors such as use of cigarette (Hakulinen et al., 2015b) and alcohol (Luchetti et al., 2018). Finally, Neuroticism is related to other aspects of health including a slower speed of walking (e.g., Stephan et al., 2018a,b) and biological dysfunctions (Sutin et al., 2019). Thus, Neuroticism may contribute to worse SRH and thus less life satisfaction.

SRH served as a significant mediator in the associations between Agreeableness and life satisfaction, Openness and life satisfaction, and Extraversion and life satisfaction. Agreeableness is positively connected to overall health (Strickhouser et al., 2017). Additionally, behaviors that benefit health including more physical activities (Artese et al., 2017), fewer addictive behaviors (Luchetti et al., 2018), and better adherence to medicine (Axelsson et al., 2011). High Openness is related to a higher level of physical activities (Sutin et al., 2016) and physical function (Stephan et al., 2018a,b) but yet a lower rate of inflammation (Luchetti et al., 2014). Extraversion is also associated with health-promoting behaviors such as more physically activities (Sutin et al., 2016; Kroencke et al., 2019), better quality of sleep (Stephan et al., 2018a,b), and better mental health (Hakulinen et al., 2015a). Furthermore, people with high Extraversion also have better objective health including a higher aerobic capacity (Terracciano et al., 2013) and better physical functions (Stephan et al., 2018a,b). Thus, high Agreeableness, Openness, and Extraversion scores may contribute to a better SRH and thus higher life satisfaction.

SRH was also a significant mediator in the connection between Conscientiousness and life satisfaction. There are positive relationships between Conscientiousness and health-promoting including physical activities (Sutin et al., 2016; Kroencke et al., 2019). However, there are also negative associations between Conscientiousness and addictive behaviors including use of alcohol (Hakulinen et al., 2015b), drug (Kang, 2022a), and cigarette (Luchetti et al., 2018; Kang, 2022b). In addition, Conscientiousness has negative relationship with chronic diseases (Weston et al., 2015) such as obesity (Jokela et al., 2013). Moreover, Conscientiousness is associated with better objective health including better lung function, more grip strength, and a faster speed of walking (e.g., Sutin et al., 2018). Finally, Conscientiousness is also linked to health marker such as metabolic, inflammatory, and cardiovascular markers (Luchetti et al., 2014; Sutin et al., 2018). Therefore, high Conscientiousness scores may contribute to a better SRH and thus higher life satisfaction.

However, all the mediations found in the current study were partial mediation and the coefficients were small, which indicates that health cannot fully explain why personality traits are associated with life satisfaction. For instance, as pointed out by Malvaso and Kang (2022), life satisfaction can also be explained by aspects including household incomes, housing, job, spouse/partner, social life, leisure time. Moreover, studies have found that personality are also related to these factors that contribute to overall life satisfaction such as social support (e.g., Yu et al., 2021). Thus, further studies on how these factors may mediate the associations between the Big Five and life satisfaction can provide a more comprehensive understanding of the underlying basis of why personality traits are related to life satisfaction. In addition, another weakness of the current research is its cross-sectional and self-reported and there is no clinical assessment of health, which may be biased. Finally, these data were quite old, which may limit its implications for today. Future studies could address these limits by including multiple mediators and using objective measurements and longitudinal designs.

## 6. Conclusion

In conclusion, SRH partially explained the relationship between the Big Five and life satisfaction on a large cohort of older adults from the UK. This study began novel exploration on if SRH could explain the connections between the Big Five and life satisfaction. Results revealed SRH could partially explain these associations in all traits. These results may offer additional support to recently developed integrated account of life satisfaction, which argues that there are no single determinants of life satisfaction. Rather, life satisfaction is made up by many factors including but not limited to personality and health (Malvaso and Kang, 2022).

The findings of this study have practical implications for interventions aimed at enhancing life satisfaction in older adults. Firstly, targeted health interventions focusing on improving self-rated health (SRH) may positively impact life satisfaction. Encouraging physical activities, reducing addictive behaviors, and improving medication adherence could be valuable strategies. Secondly, considering the influence of personality traits on SRH and life satisfaction, interventions that foster traits including Agreeableness, Openness, Conscientiousness, and Extraversion but reduce Neuroticism may have a positive effect. Lastly, a comprehensive approach that acknowledges the multidimensional nature of life satisfaction, including personality, health, and other life domains such as income, housing, and social support, is crucial for promoting overall well-being in older adults. By incorporating these strategies, practitioners and policymakers can design interventions that address the complex interplay of factors contributing to life satisfaction in this population.

## Data availability statement

Publicly available datasets were analyzed in this study. This data can be found at: https://www.understandingsociety.ac.uk.

## Ethics statement

The studies involving human participants were reviewed and approved by University of Essex. The patients/participants provided their written informed consent to participate in this study.

## Author contributions

WK: conceptualization, data curation, formal analysis, investigation, methodology, resources, software, supervision, writing—original draft, and writing—review and editing. AM: writing—original draft. All authors contributed to the article and approved the submitted version.

## Conflict of interest

The authors declare that the research was conducted in the absence of any commercial or financial relationships that could be construed as a potential conflict of interest.

## Publisher’s note

All claims expressed in this article are solely those of the authors and do not necessarily represent those of their affiliated organizations, or those of the publisher, the editors and the reviewers. Any product that may be evaluated in this article, or claim that may be made by its manufacturer, is not guaranteed or endorsed by the publisher.

## Footnotes

^1^https://github.com/canlab/MediationToolbox
