# Supplementary material for: Self-rated health (SRH) partially mediates and associations between personality traits and life satisfaction in older adults
Source: Front Psychol. 2023 Jul 6;14:1189194. doi: 10.3389/fpsyg.2023.1189194 (PMC10359495; doi:10.3389/fpsyg.2023.1189194)
Supplement: Supplementary file 1 [file Table_1.docx]

Supplementary material

Table.1. Questions in the 15-item version of the Big Five for measuring personality traits.

| Neuroticism | I see myself as someone who worries a lot. |
| --- | --- |
|  | I see myself as someone who gets nervous easily. |
|  | I see myself as someone who is relaxed, handles stress well. |
| Agreeableness | I see myself as someone who is sometimes rude to others. |
|  | I see myself as someone who has a forgiving nature. |
|  | I see myself as someone who is considerate and kind to almost everyone. |
| Openness | I see myself as someone who is original, comes up with new ideas. |
|  | I see myself as someone who values artistic, aesthetic experiences. |
|  | I see myself as someone who has an active imagination. |
| Conscientiousness | I see myself as someone who does a thorough job. |
|  | I see myself as someone who tends to be lazy. |
|  | I see myself as someone who does things efficiently. |
| Extraversion | I see myself as someone who is talkative. |
|  | I see myself as someone who is outgoing, sociable. |
|  | I see myself as someone who is reserved. |
